# Utilization of Adipose-Derived Stem Cells in Cranial Nerve Regeneration: A Comprehensive Review

**DOI:** 10.7759/cureus.88706

**Published:** 2025-07-24

**Authors:** Nolan Brown, Saarang Patel, Mohammad F Khan, Ryan T Gensler, Hibbah I Khan, Vikas Munjal, Julian L Gendreau, Taylor Reardon

**Affiliations:** 1 Neurological Surgery, University of California, Irvine, Irvine, USA; 2 Neurosurgery, Seton Hall University, South Orange, USA; 3 Neurosurgery, Indiana University School of Medicine, Indianapolis, USA; 4 Neurosurgery, Georgetown University School of Medicine, Washington, USA; 5 Neurosurgery, The Ohio State University, Columbus, USA; 6 Biomedical Engineering, Johns Hopkins University, Baltimore, USA; 7 Neurology, Henry Ford Health, Detroit, USA

**Keywords:** adipose-derived stem cell, cranial nerve, neurosurgery, peripheral nerve regeneration, stem cell

## Abstract

Large peripheral nerve injuries may require surgical reconnection. Cell-based therapies have also been investigated for nerve regeneration. Within the context of this modern treatment paradigm for peripheral nerve injuries, we investigated the role of adipose-derived stem cells (ADSCs) in the regeneration of cranial nerves. PubMed and Embase databases were used to search for primary studies reporting the use of ADSCs in the regeneration of cranial nerves. A total of 12 studies were included, all of which presented data on specific neural injury, therapy, and functional outcomes. Eight studies focused on the facial nerve (66.7%), two on the optic nerve (16.7%), one on the olfactory nerve (8.3%), and one on the hypoglossal nerve (8.3%). One study applied ADSCs to human cranial nerve injuries, while the remainder studied animal models. In these studies, ADSC groups had higher numbers of myelinated fibers, increased myelin thickness, and diameter of muscle fibers, as well as greater magnitude of compound muscle action potentials (CMAP) when compared to controls. In studies focused on optic nerve regeneration, significant improvements across visual tests were observed. ADSCs demonstrate potential utility in regard to their ability to facilitate functional recovery of cranial nerves in humans and animal models. As such, this therapy merits further investigation so that its true clinical applications can be determined.

## Introduction and background

Peripheral nerve injuries are potentially devastating and disproportionately affect young healthy individuals [1]. Such injuries most frequently result from either traumatic nerve transection or crush injuries [2,3]. Less common etiologies include birth-related trauma, ischemic nerve injury, and tumor invasion [2,3]. Peripheral nerve injuries can have profound consequences for patients, such as reduced quality of life and an increased socioeconomic burden due to reduced capacity to work [4,5].

While peripheral nerve axons have intrinsic regenerative capacity and can form functional connections with their target tissue, a highly specialized environment is required to support this process. After the initial nerve injury, the terminal axon segment distal to the site of injury undergoes Wallerian degeneration [3]. Schwann cells (SCs), critical components of the nerve regeneration process, proliferate and line the damaged axon segments to support regrowth of the viable nerve stump. SCs also upregulate growth-associated genes and secrete neurotrophic growth factors and cytokines, which recruit macrophages to phagocytize lysed myelin segments [2,3].

The intrinsic axon regenerative capacity of peripheral nerves may be insufficient in injuries with a large nerve gap defect (e.g., >1 cm). Large nerve gaps prevent distal nerve fibers from contacting target tissue for a sufficient duration to cause SC atrophy, which impairs their critical role in peripheral nerve regeneration and compromises functional recovery [3,6-8]. In these cases, there are several treatment options. Surgical reconnection of peripheral nerves, known as "coaptation," has long been a viable option for nerve gap defects up to one centimeter, as the reconstructive surgeon can reconnect the proximal and distal nerve endings without causing significant tension on the nerve. When undue tension precludes primary nerve reconstruction, autologous nerve grafting is performed; in fact, this is the gold standard for repair of large nerve gap defects [3,5,9]. Autologous nerve grafts may be harvested from multiple sites (e.g., sural nerve, lateral antebrachial cutaneous nerve, dorsal cutaneous branch of the ulnar nerve, etc.), are nonimmunogenic, and studies have indicated their efficacy in repairing nerve gap defects as large as 10-20 cm [3,10]. However, there are also considerable disadvantages to this method. These include the potential for donor site morbidity, cosmetic deformity, requirement for two anastomotic sites, increased operative time, and limited graft supply if the nerve repair requirement exceeds donor capacity [9,11-13]. As such, there has been an ongoing search for a viable alternative option.

Several cell-based therapies have been developed and investigated for use in peripheral nerve regeneration. Of particular interest are adipose-derived stem cells (ADSCs), which are multipotent mesenchymal stromal cells that have the capacity to differentiate into multiple mesodermal cellular phenotypes. High quantities of ADSCs are easily harvested using minimally invasive liposuction procedures, demonstrate high proliferative rates in vitro, and can differentiate into SC-like cells and secrete a variety of neurotrophic factors, which may augment peripheral nerve regeneration [3,12-15].

Many studies have demonstrated the efficacy of utilizing ADSCs for peripheral nerve regeneration, both in vivo and in vitro. In this review, we focus on studies specifically investigating the use of ADSCs for cranial nerve regeneration, as these nerves play a critical role in our ability to function. We aim to present the current research regarding the use of ADSCs for cranial nerve regeneration using in vivo models and discuss future directions for research.

## Review

Methods

Search Strategy

A search was completed using both the Medline PubMed and Embase databases. All potential included studies were identified with search terms, including “peripheral nerve”, “repair”, “regeneration”, “CRISPR”, “Cas-9”, and “adipose stem cell”. This search included all studies published from January 2000 to April 2022 [16].

Review Process

Duplicates were removed after completing the initial searches. All abstracts were collected and reviewed independently by two authors. Any disagreements in inclusion or exclusion were resolved by a third author. After this was completed, full texts of the remaining studies were retrieved and reviewed for final inclusion by the same authors.

Studies were included if they were focused on the regeneration of cranial nerves in either humans or animal models. Studies were excluded if they were in vitro studies, review articles (systematic review/meta-analysis), not a full study (abstracts/conference papers/editorials/narrative reviews), not originally published in English, intervention that was not ADSC, either undifferentiated or differentiated, or CRISPR Cas-9, not focused on the cranial nerves, no reported findings of a nerve model, no reported findings of nerve regeneration, case reports, or not had full texts available. Final included studies then underwent a data collection process that involved collecting which cranial nerve was investigated, type of animal used, differentiation status of ADSC, number of specimens in treatment groups, number of myelinated fibers, muscle fiber area, muscle fiber diameter, myelin thickness, g-ratio (defined as ratio between the inner axon radius and the outer, myelinated, axon radius), facial palsy score, compound muscle action potential (CMAP), identified contributory genes or proteins, visual performance score, visual acuity change, microperimetry sensitivity, best corrected visual acuity, Vibrissae motor performance, and whisker motion outcomes. 

Results

Study Demographics

A total of 12 studies met the inclusion criteria. Of these, eight (66.7%) were focused on the facial nerve [13,17-23], two (16.7%) on the optic nerve [24,25], one (8.3%) on the olfactory nerve [26], and one (8.3%) on the hypoglossal nerve [27]. There was one human study that focused on optic nerve regeneration [25]. All other studies used animal models, of which there were nine (83.4%) rat models [13,17-19,21-24,26], one (8.3%) rabbit model [27], and one (8.3%) dog model [20]. Eight (66.7%) of these papers focused on the regeneration of cranial nerves using undifferentiated ADSC [17,18,20-22,24,25,27], two (16.7%) studied only differentiated ADSC (dADSC) [19,26], and two (16.7%) involved both ADSC and dADSCs [13,23]. A summary of all findings is presented in Table 1.

**Table 1:** Overview of the included studies ADSC: adipose-derived stem cells; dADSC: diferrentiated adipose-derived stem cells

Authors/Year	Cranial Nerve	Model Used	Type of ADSC	Key Outcomes Assessed	Functional Tests	Key Results	Additional Notes
Watanabe et al. (2017) [13]	Facial nerve	Rat (7 mm defect model)	Undifferentiated and differentiated ADSCs	Number of myelinated fibers, myelin thickness, nerve fiber area, g-ratio (axon-to-fiber diameter ratio)	Rat Facial Palsy Scoring System (FPS)	Both uADSCs and dADSCs significantly improved nerve regeneration similar to Schwann cells and close to autograft controls; improved FPS significantly by week 6 and reached autograft scores by week 12; uADSCs: 2386 fibers, 0.51 µm myelin thickness; dADSCs: 2544 fibers, 0.54 µm thickness	Undifferentiated ADSCs (uADSCs) maintained mesenchymal characteristics (Stro-1 positive); Differentiated ADSCs (dADSCs) expressed Schwann cell markers (S100β, NGFp75R, GFAP). No tumorigenesis or neuroma formation reported
Abbas et al. (2016)​ [17]	Facial nerve (cross-face graft)	Rat (cross-facial nerve graft with sciatic nerve interposition)	Undifferentiated ADSCs	Fiber density, myelin thickness, fiber diameter, g-ratio, neuromuscular junction (ACh)	Vibrissae movement (biometric, electrophysiologic assessments)	ADSCs significantly enhanced axonal regeneration, improved fiber density and myelin thickness, and acetylcholine expression at neuromuscular junctions	ADSCs locally injected along the graft line significantly enhanced facial nerve regeneration
Fujii et al. (2020)​ [18]	Facial nerve (cross-face graft)	Rat (facial palsy model, sciatic nerve graft)	ADSC suspension vs. ADSC sheets	Time to reinnervation, CMAP amplitude, number of myelinated fibers	Facial Palsy Scoring System (FPS), CMAP	ADSC sheets significantly accelerated reinnervation (4.2 mV amplitude, 2450 fibers); suspension (1.7 mV, 1645 fibers), control (1.6 mV, 1049 fibers)	ADSC sheets were superior to ADSC suspensions, promoted faster axonal outgrowth and better physiological recovery
Fujimaki et al. (2019)​ [19]	Facial nerve	Rat (7 mm defect)	Dedifferentiated fat cells (DFAT)	Number of myelinated fibers, myelin thickness, CMAP amplitude, whisker motion angle	Whisker motion assessment, CMAP	DFAT conduits had significantly more fibers (1605 vs. 544), thicker myelin (0.57 µm vs. 0.46 µm), greater whisker motion (9.22° vs. 1.9°); CMAP amplitude not significantly different (2.84 mV vs. 0.88 mV)	DFAT cells embedded in PGA-collagen conduits improved structural and functional recovery
Ghoreishian et al. (2013)​ [20]	Facial nerve	Dog (7 mm nerve gap)	Undifferentiated ADSCs encapsulated in alginate hydrogel	Axon counts, nerve diameter, nerve conduction velocity (NCV), maximal action potential amplitude	NCV, CMAP	ADSCs significantly improved nerve conduction velocity (28.5 m/s vs. 16.2 m/s) and CMAP amplitude (1.86 mV vs. 1.45 mV); axon counts similar (67% of normal nerve)	Alginate-encapsulated ADSCs enhanced functional nerve regeneration despite similar histological results compared to controls
Shimizu et al. (2018)​ [21]	Facial nerve	Rat (7 mm defect)	ADSCs vs. Stromal Vascular Fraction (SVF)	CMAP amplitude, axon diameter, myelin thickness, fiber diameter	CMAP	SVF group had the largest axon diameter, fiber diameter; the ADSC group had the greatest myelin thickness; both groups were superior to the control (empty conduits)	SVF more practical than ADSCs due to rapid preparation, but ADSCs demonstrated superior myelination
Sun et al. (2011a)​ [22]	Facial nerve	Rat (8 mm buccal branch defect)	Undifferentiated ADSCs in decellularized artery conduit	Axonal growth, myelinated fiber maturation, facial motoneuron labeling	Vibrissae movement scale	ADSCs improved axonal growth and whisker function significantly over empty conduits but inferior to nerve autografts	Decellularized artery conduit effectively supported ADSC integration and improved nerve regeneration
Sun et al. (2011b) [23]​	Facial nerve	Rat (8 mm buccal branch defect)	Transdifferentiated ADSCs (dADSCs) in decellularized artery conduit	Axon growth, CMAP amplitude, retrograde facial motoneuron labeling, number of myelinated fibers	Vibrissae movement, CMAP	dADSC-seeded conduits showed significantly superior regeneration compared to undifferentiated ADSCs and comparable to Schwann cell-seeded conduits; nerve autografts remained superior overall	dADSCs maintained Schwann-cell-like phenotype, effectively supported axon regeneration and remyelination
Li et al. (2018)​ [24]	Optic nerve	Rat (optic nerve crush model)	Undifferentiated ADSCs	Retinal ganglion cell (RGC) count, GAP-43 mRNA expression, apoptosis markers	N/A	ADSCs transplantation significantly increased RGC survival and GAP-43 mRNA expression; reduced retinal apoptosis compared to controls	Transplanted ADSCs exhibited neuroprotective effects, promoting neuronal survival and regeneration after optic nerve injury
Limoli et al. (2021)​ [25]	Optic nerve (Glaucomatous optic neuropathy)	Human clinical study	Autologous ADSCs (in stromal vascular fraction with PRP) grafted suprachoroidally	Best-corrected visual acuity (BCVA), close-up visual acuity, retinal sensitivity by microperimetry, OCT	Visual acuity tests, microperimetry	Significant improvements in BCVA, near-vision, and microperimetric sensitivity after ADSC graft; functional improvements significantly greater than untreated controls	Clinical demonstration of ADSC effectiveness; highlights neurotrophic factor secretion and paracrine regenerative mechanisms
Kim et al. (2009)​ [26]	Olfactory nerve	Rat (olfactory nerve transection)	Undifferentiated ADSCs (systemic injection)	Olfactory marker protein (OMP) expression, epithelial thickness, cell proliferation (PCNA)	None (histological only)	ADSCs promoted regeneration of olfactory epithelium, restored epithelial thickness, increased PCNA+ cells, and showed partial differentiation into olfactory neurons and endothelial cells	Intravenous ADSCs migrated to the olfactory epithelium and survived ≥4 weeks; supported regeneration likely via paracrine and differentiation effects
Wada et al. (2021)​ [27]	Hypoglossal nerve	Rabbit (nerve transection with tongue atrophy)	ADSCs mixed with fat tissue (local injection)	Inferior longitudinal muscle fiber area and diameter, Pax7+ cell expression	Food intake, body weight (no specialized motor testing)	ADSCs + fat significantly increased muscle fiber area (582±312 µm² vs. 405±220 in control) and diameter (24±8 µm vs. 20±6); enhanced Pax7+ satellite cell recruitment	Demonstrated safety and feasibility of ADSC-enhanced fat injection for muscle regeneration post-denervation; no adverse effects observed

Number of Myelinated Fibers

Five (41.7%) studies on the facial nerve utilized a qualitative approach to analyze the success of nerve regeneration in cranial nerve based on the number of myelinated fibers after a period of time after treatment with ADSCs [13,18-21]. All studies found an increase more myelinated fibers after ADSC intervention (range across all studies was 1,026-2,736 fibers) compared to the controls (range: 543-1,700 fibers) [13,18-21]. Both studies that used dADSCs reported similar findings (range: 1,606-2,544 fibers vs. 543-1,449) [13,19]. A single study focusing on facial nerve regeneration reported a greater improvement in the number of myelinated fibers in the group treated with dADSC compared to the ADSC (2544±130.2 vs. 2386±221 fibers) [13].

Myelin Thickness

Six (50.0%) studies on the facial nerve reported the change in myelin thickness [13,18,19,21-23]. One study did not report quantitative data but did not find a significant improvement in either the ADSC or dADSC groups when compared to the control group [23]. Three studies reported that the ADSC intervention (range: 0.51-0.80 mm) increased the mean myelin thickness compared to the controls (range: 0.40-0.60 mm) [18,21,22]. Another study found greater myelin thickness in dADSC compared to the control group (0.57 vs. 0.46 mm; p<0.01). There was only one study that reported the myelin thickness in both the ADSC and dADSC groups, in which they found a slightly increased value in the ADSC group (0.54 vs. 0.51 mm) [13].

G-Ratio

The G-ratio is the ratio between the inner axon radius and the outer, myelinated axon radius. It was reported in five (41.7%) studies that focused on the facial nerve [13,17,19,21,23]. The g-ratio in the ADSC groups was found to be insignificantly different by two studies [21,23], increased in ADSC groups by one study [17], and decreased in one study [13]. Two studies reporting on facial nerve regeneration found conflicting results; one found no difference in the g-ratio means of the dADSC compared to the control, whereas the other found a slight decrease in g-ratio [13].

Compound Muscle Action Potential (CMAP)

The CMAP was reported by six (50.0%) studies on facial nerve regeneration. All studies found increased values of CMAP in both ADSC (range: 1.1-5.37 mV) [17,18,20,21,23] and dADSC groups (2.8 mV) [19], compared to the controls (range: 0.70-3.11 mV) [17-21,23]. 

Muscle Fiber Changes 

A total of six (50.0%) studies reported the muscle fiber diameter; one focused on the hypoglossal nerve [27] and the remaining studied the facial nerve [18-21,23]. One study did not report quantitative data in their writing, but concluded through graphs that the sham, nerve autograft (AG), Schwann cells (SC), and dADSC groups all increased significantly compared to the control group [23]. Several other studies focusing on the facial nerve reported the diameter as a measurable value and demonstrated an increase in the ADSC group (range: 1.90-5.70 mm) compared to the control (range: 1.90-5.08 mm) [18-21]. In the lone study analyzing the effect of ADSC on hypoglossal nerve regeneration, the authors found an improvement in ADSC compared to controls (24 vs. 20 mm; p<0.05). The authors also reported greater muscle fiber area in the ADSC group compared to the control (582 vs. 405 mm^2^; p<0.05) [27]. This finding was corroborated by one study looking at muscle fiber area in facial nerve regeneration in patients treated with ADSCs vs. control (5.69 vs. 3.43 mm^2^; p<0.001) [13].

Nerve Functionality Improvement After Regeneration

Each cranial nerve has unique evaluative measures due to the diverse functionality of these nerves. Facial nerve function was assessed using the Vibrissae Motor Performance score in three studies, all of which found significant improvement in scores of the ADSC group compared to controls [17,22,23]. Another tool used for facial nerve functionality analysis is the facial palsy scoring system (FPS). Both studies reporting through this method found significantly increased scores in the ADSC group compared to controls [13,18]. One rodent study compared improvement in whisker motion after facial nerve transection in mice/rats treated with dADSC compared to controls, and there were significant improvements in the dADSC cohort (9.22 vs. 1.90 degrees; p<0.01) [19].

One study on optic nerve injury in humans treated with ADSC vs. control found significantly improved values in the ADSC group in microperimetry sensitivity (+11.24% vs. -4.24%; p=0.003) and best corrected visual acuity (+27.32% vs. +1.09%; p=0.02) [25]. 

Indirect Measurements of Regeneration

Two studies reported indirect measurements of regeneration: one animal study focusing on the optic nerve, and another on the olfactory nerve [24,26]. The former reported that higher levels of growth-associated protein-43 (GAP43) mRNA were identified in early recovery of optic nerve damage in groups treated with ADSCs compared to controls [24]. They also found that ADSC treatment was associated with significantly higher B-cell lymphoma-2 (Bcl-2) protein levels. As Bcl-2 is an established anti-apoptotic protein, this suggests that ADSC treatment may promote optic nerve cell survival. The latter study reported that after olfactory nerve transection and supplementation with ADSC, the olfactory epithelium (innervated by the olfactory nerve) had returned to its original thickness by 30 days after surgery [26].

Discussion

To identify a methodology to maximize nerve regeneration after injury, researchers have taken a multidisciplinary approach involving gene/protein identification studies, animal models, and even some early experiences in humans. Both differentiated and undifferentiated ADSCs have been heavily investigated, and preliminary results indicate the ability to improve nerve regeneration, along with significant recovery in cranial nerve function. To the authors’ knowledge, no previous study has reviewed outcomes of ADSC and dADSC in cranial nerve regeneration. To fill this gap in the literature, the current study identified and described 12 studies focused on the effect of ADSC/dADSC treatment on regeneration, muscle recovery, and functional recovery of various cranial nerves. Although the literature on ADSCs is primarily animal research, we found that ADSC treatment was associated with improvements in several variables indicative of remyelination and muscle innervation. This suggests a potential role for ADSCs in augmenting the repair of cranial nerve injuries.

Nerve Fiber Characteristics

Our review found that electrodiagnostic studies, such as nerve conduction velocity (NCV), electromyography (EMG), and CMAP showed improvement in cranial nerve injury models treated with ADSCs compared to controls. Nerve conduction studies and EMG have long been the gold standard for evaluation of peripheral nerves and cranial nerves and their response to stimulus [28]. Responses to EMG/NCV describe the functionality of the individual nerves; therefore, these evaluative tools can be utilized to quantify the amount of nerve regeneration after injury. Ikeda et al. published a study that analyzed NCV and the morphological changes of axonal fibers [29] and reported that the motor NCV results correlated well with mean fiber diameter [29]. We found that all six of the studies that reported the change in myelinated fiber thickness saw a greater improvement in cohorts treated with ADSC or dADSC than controls [13,18,19,21-23]. CMAP is another parameter utilized to evaluate nerve functionality, and it was found to increase in both the ADSC and dADSC groups [17-21,23].

The g-ratio was reported by five studies included in this review, but the findings varied greatly. The g-ratio in animals treated with ADSC was found to be insignificantly different from the control group in two studies [21,23], increased in one study [17], and decreased in another [13]. One study did, however, find poor correlation between the g-ratio and NCV and concluded that the g-ratio may not be an accurate predictor of nerve recovery [29].

Muscle Fiber Characteristics

Another approach to evaluate nerve regeneration is to identify the muscles innervated by the individual cranial nerves and look at the muscle fibers of them. Since muscles that lose their innervation eventually become atrophied and lose many of their normal characteristics, structural changes in muscle tissue can be tracked and analyzed as a function of nerve regeneration [2]. In the current study, muscle fiber diameter [18-21,23,27] and muscle fiber area [27] were both found to be improved greatly in both the ADSC and dADSC groups compared to controls. When the muscle improvements are paired with increased myelin thickness and increased number of myelinated fibers, they collectively support the use of ADSC and dADSC as valid interventions for improved cranial nerve regeneration.

Evaluative Tools for Nerve Regeneration

With each cranial nerve serving unique functions, there is no singular method to evaluate their state of health. The facial nerve can be assessed through the Vibrissae Motor Performance score, FPS, and degree of whisker motion. All studies found significant improvements in the functioning of the facial nerve in both the ADSC and dADSC groups across a variety of these scoring systems [13,17-19,22,23]. The optic nerve is evaluated using microperimetry sensitivity and best corrected visual acuity, and the lone study on optic nerve regeneration found improvements in each functional test in the ADSC-treated eyes when compared to controls [25]. The literature did not provide a consistent evaluative tool for either the hypoglossal or olfactory nerves. The olfactory nerve is difficult to evaluate because there is sense of smell is subjective and may differ from patient to patient. However, because the olfactory epithelium regenerates due to the presence of nerve signals from the olfactory nerve, olfactory epithelial thickness is a reasonable proxy for nerve regeneration. The lone study on olfactory epithelial thickness demonstrated complete recovery after 30 days in the ADSC groups [26].

Limitations and Future Directions

The main limitation of this study is that all included studies, except one, reported the outcomes of ADSC/dADSC supplementation for peripheral nerve regeneration in animal models. The one study that was completed in humans demonstrated significant regeneration of the optic nerve after being supplemented with ADSCs. This study demonstrated a 27-fold percent improvement in best corrected visual acuity (+27.32% vs. +1.09%; p=0.02) and a significant increase in microperimetry (+11.24% vs. -4.24%; p=0.003), when compared to control groups. The success of this study may promote further investigation into the use of ADSCs as an aid for cranial nerve regeneration in humans.

## Conclusions

The use of ADSCs and dADSCs has long been investigated as a potential treatment for cranial and peripheral nerve regeneration. This review demonstrates that the use of ADSC improves the recovery of nerves, muscles, and functionality of the cranial nerves. Further investigation into the use of ADSCs should be pursued in both animal and early human studies to validate the findings of previous literature.
